# Impaired Redox Signaling in Huntington’s Disease: Therapeutic Implications

**DOI:** 10.3389/fnmol.2019.00068

**Published:** 2019-03-19

**Authors:** Bindu D. Paul, Solomon H. Snyder

**Affiliations:** ^1^The Solomon H. Snyder Department of Neuroscience, Johns Hopkins University School of Medicine, Baltimore, MD, United States; ^2^Department of Psychiatry and Behavioral Sciences, Johns Hopkins University School of Medicine, Baltimore, MD, United States; ^3^Department of Pharmacology and Molecular Sciences, Johns Hopkins University School of Medicine, Baltimore, MD, United States

**Keywords:** Huntington’s disease, oxidative stress, redox signaling, cysteine, mitochondria

## Abstract

Huntington’s disease (HD) is a neurodegenerative disease triggered by expansion of polyglutamine repeats in the protein huntingtin. Mutant huntingtin (mHtt) aggregates and elicits toxicity by multiple mechanisms which range from dysregulated transcription to disturbances in several metabolic pathways in both the brain and peripheral tissues. Hallmarks of HD include elevated oxidative stress and imbalanced redox signaling. Disruption of antioxidant defense mechanisms, involving antioxidant molecules and enzymes involved in scavenging or reversing oxidative damage, have been linked to the pathophysiology of HD. In addition, mitochondrial function is compromised in HD leading to impaired bioenergetics and elevated production of free radicals in cells. However, the exact mechanisms linking redox imbalance to neurodegeneration are still elusive. This review will focus on the current understanding of aberrant redox homeostasis in HD and potential therapeutic interventions.

## Introduction

Huntington’s disease (HD) is an autosomal dominant neurodegenerative disease caused by expansion of CAG repeats in the gene huntingtin, *htt*, present on chromosome 4 (MacDonald et al., 1993). About 1 in 7500 individuals are affected by HD worldwide, and no satisfactory cure exists so far (Fisher and Hayden, 2014). HD primarily affects the corpus striatum of the brain and manifests as abnormal involuntary movements, motor and cognitive deficits (Bates et al., 2015; McColgan and Tabrizi, 2018). Once symptoms appear, median survival is about 18 years (Ross et al., 2014). The number of CAG repeats in mutant htt (mHtt) inversely correlates with the age of onset of disease (Illarioshkin et al., 1994; Brandt et al., 1996). Translation of the CAG repeats results in an abnormally long polyglutamine repeat at the N-terminal end of Htt. mHtt is proteolytically cleaved and the N-terminal fragments aggregate to form inclusion bodies, a characteristic feature of the disease, although its role in disease progression is debated (Arrasate and Finkbeiner, 2012). Immunostaining with antibodies against mHtt led to the discovery of intranuclear inclusions in mouse models and patient samples, a hallmark of the disease (Davies et al., 1997; DiFiglia et al., 1997). mHtt impacts multiple cellular processes ranging from transcriptional and translational regulation, mitochondrial function, DNA replication and repair to nucleocytoplasmic transport which leads to neurotoxicity (Gu et al., 1996; Luthi-Carter et al., 2002; Lu X.H. et al., 2014; Banez-Coronel et al., 2015; Grima et al., 2017; Maiuri et al., 2017).

A prominent feature of HD is elevated oxidative stress. Oxidative stress is usually defined as a balance of prooxidant-antioxidant tendencies favoring the former. However, with advances in the field, oxidative stress is more appropriately defined as a disruption of redox signaling (Jones, 2006). Physiological levels of oxidative stress may be important for cellular processes (eustress) but excess, uncontrolled oxidative stress may be deleterious (distress) (Sies et al., 2017). The brain is particularly vulnerable to damage induced by oxidative stress. The brain is amongst the most metabolically active organ in the body and accounts for about 20% of the oxygen consumed (Gadoth and Goebel, 2011). The lipid-rich composition of the brain with its suboptimal antioxidant defense mechanisms as compared to peripheral tissues makes it a target for free radical-induced damage (Floyd and Carney, 1992). Reactive oxygen, nitrogen and sulfur species (ROS, RNS, and RSS) can induce protein oxidation, lipid peroxidation and DNA damage (Sbodio et al., 2018b). As in HD, oxidative stress has been observed in other neurodegenerative disorders including Alzheimer’s disease (AD), Parkinson’s disease (PD), Amyotrophic lateral sclerosis (ALS) and Ataxias and is linked to pathogenesis (Sbodio et al., 2018b).

## Redox Imbalance and HD

**FIGURE 1:**
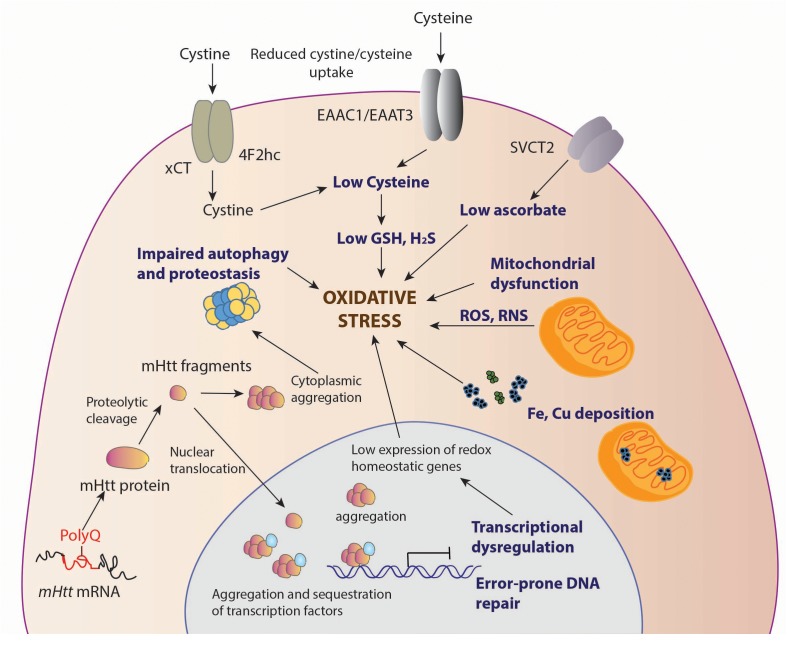
Sources of oxidative stress in HD. Decreased levels of antioxidants, cysteine and ascorbate is observed in HD. The cysteine transporter, excitatory amino acid transporter 3 (EAAT3/EAAC1) is dysregulated in HD leading to decreased intake of cysteine. The uptake of the oxidized form of cysteine, cystine, mediated by the ${\text{x}}_{\text{c}}^{-}$ system, composed of the light chain xCT, and the heavy chain 4F2hc, is also reduced, contributing to low cysteine levels. Ascorbate influx via the SVCT2 transporter is also limited in HD, which leads to reduced antioxidant defense in neurons. Deposition of transition metals such as iron (Fe) has been observed both in the cytoplasm and mitochondria, leading to elevated levels of free radicals which can damage cellular components. Mutant huntingtin (mHtt) aggregates both in the nucleus and cytoplasm affecting multiple cellular processes, which include mitochondrial function, autophagy and proteostasis, which leads to elevated oxidative stress. In the nucleus, mHtt sequesters or affects transcription factors, several of which are involved in regulation of antioxidant defense mechanisms, further contributing to redox imbalance in cells. mHtt also affects DNA repair processes, which results in error prone repair and damage.

Several studies have reported oxidative damage in cells and tissues from HD models and patient samples. Elevated markers of damage such as protein oxidation, lipid peroxidation, and DNA damage have been linked to HD. The damage could stem from a variety of abnormalities arising due to the toxic effects of mHtt (summarized in Figure 1).

### Deposition of Metal Ions

Metal ions such as iron (Fe), copper (Cu), manganese (Mn), and Zinc (Zn) serve as cofactors for a variety of enzymes and participate in processes such as electron transport, redox regulation, and oxygen transport among others. These metals are beneficial in trace amounts, but excess accumulation leads to several pathological conditions. Iron is redox active, existing in the ferrous (Fe^2+^) and ferric (Fe^3+^) states. The Fe^2+^ form participates in the Fenton reaction reacting with hydrogen peroxide (H_2_O_2_) to generate the highly reactive hydroxyl radical (**.**OH) and HO_2_, which can cause oxidative damage to cellular components. Elevated iron content has been observed in the basal ganglia in symptomatic and late stage HD (Bartzokis et al., 1999, 2007; Bartzokis and Tishler, 2000). Iron accumulates in both neurons and glia, and treatment with deferoxamine, an iron chelator, affords neuroprotection in the R6/2 mouse model of HD (Simmons et al., 2007; Chen et al., 2013). Conversely, iron supplementation in the diet of neonatal R6/2 mice promotes neurodegeneration in the R6/2 mice (Berggren et al., 2015). Neonatal iron supplementation resulted in iron accumulation in mitochondria due to the increased expression of the mitochondrial iron transporter mitoferrin 2 (Agrawal et al., 2018). In addition to iron, excess copper deposition also mediates neurodegeneration in HD (Dexter et al., 1992; Fox et al., 2007). Copper binds the N-terminal region of mHtt, promotes its aggregation and delays its clearance (Fox et al., 2011). Accordingly, therapies preventing the accumulation of these redox active metals may prove beneficial.

### Altered Levels of Antioxidant Molecules and Enzymes

Cells harbor an array of metabolites and molecules that counteract oxidative damage. These may be endogenously synthesized or obtained from the diet. Diminished levels of the antioxidants cysteine, glutathione (GSH), coenzyme Q_10_ (CoQ_10_) and ascorbate have been observed in HD and could potentiate disease progression (Andrich et al., 2004; Paul et al., 2014).

#### Vitamin C/Ascorbate

Vitamin C/ascorbate is a water soluble molecule and cofactor for several enzymatic processes, which regulates metabolism and protects neurons against oxidative stress (Padh, 1990; Castro et al., 2009). During neuronal activity, glutamate is taken up and ascorbate released by astrocytes, which is accumulated by neurons via a specific transporter, SVCT2 (Wilson et al., 2000; Castro et al., 2001). Neuronal ascorbate promotes utilization of lactate over glucose during synaptic activity and also modulates redox balance. The uptake of ascorbate was compromised in cell culture and R6/2 mouse models of HD due to impaired translocation of SVCT2 to the plasma membrane and these changes preceded mitochondrial dysfunction (Acuna et al., 2013). Supplementation of ascorbate reversed the deficits.

#### Cysteine

Cysteine is a semi-essential amino acid which is synthesized endogenously as well as obtained from the diet. The availability of cysteine is the rate limiting step for glutathione biosynthesis. We have shown previously that cysteine metabolism is compromised in HD (Paul et al., 2014, 2018). Expression of the biosynthetic enzyme for cysteine, cystathionine γ-lyase (CSE) is drastically decreased in HD due to the sequestration of its transcription factor, specificity protein1 (SP1) by mHtt. SP1 regulates transcription of CSE during basal conditions. During stress, expression of CSE is controlled by the stress-responsive activating transcription factor 4 (ATF4). In HD cells, induction of ATF4 is also suboptimal leading to decreased CSE expression and cysteine biosynthesis during stress (Sbodio et al., 2016). Both the biosynthesis and uptake of cysteine and its oxidized form are impaired in HD. Activity of the neuronal cysteine transporter, EAAT3/EAAC1 is decreased in HD due to inhibition of its trafficking to the plasma membrane (Li et al., 2010). Cysteine exists as its oxidized form, cystine, in extracellular fluids and is taken up by the cystine transport system, ${\text{x}}_{\text{c}}^{-}$ system, composed of the light-chain (xCT, encoded by the *SLC7A11* gene and the heavy-chain subunit 4F2hc, encoded by the *SLC3A2* gene) into cells, where the reducing atmosphere converts it to its monomeric, reduced form, cysteine. The activity of xCT is decreased in HD leading to suboptimal cystine metabolism (Frederick et al., 2014). This is not surprising, considering that ATF4 is one of the transcription factors for the transporter’s expression. Supplementing cysteine and its stable precursor, *N*-acetyl cysteine (NAC) in the diet of R6/2 mice alleviated some of the symptoms and delayed disease progression. Cysteine is also the substrate for the generation of hydrogen sulfide (H_2_S), a gaseous signaling molecule that participates in a myriad of physiological processes. Three enzymes, CSE, cystathionine β-synthase (CBS) and 3-mercaptopyruvate sulfur transferase (3-MST), utilize cysteine to generate H_2_S. H_2_S signals by sulfhydration also called persulfidation, wherein the reactive –SH group of a cysteine residue is converted to a persulfide or –SSH group (Mustafa et al., 2009). Sulfhydration regulates the activity of several proteins and thus modulates several signal transduction cascades (Paul and Snyder, 2012, 2015a,b, 2018). As cysteine metabolism and CSE expression are altered in HD, levels of H_2_S are diminished in HD. We demonstrated that upregulating the transsulfuration pathway to induce CSE expression and H_2_S production has therapeutic benefits (Sbodio et al., 2018a,c). Cysteine is utilized by cells to generate several sulfur containing molecules such as cystamine, taurine, lanthionine, homolanthionine, and coenzyme A. The metabolism of these molecules in HD remain to be investigated.

#### Glutathione

Glutathione, a tripeptide composed of glutamate, cysteine and glycine, is a major antioxidant involved in maintenance of redox homeostasis in cells (Meister and Anderson, 1983). GSH metabolism is dysregulated in HD, which could contribute to the redox imbalance observed in HD. Plasma GSH levels are inversely correlated to caudate atrophy in HD patients (Pena-Sanchez et al., 2015). Decrease in GSH in the postmortem cortex of HD patients has also been observed (Beal et al., 1992). However, other studies have detected increased intracellular glutathione levels in cell culture HD models and increased GSH in cortical and striatal mitochondria of the R6/2 mouse model of HD, although this increase was not sufficient to alleviate the oxidative stress associated with the disease (Choo et al., 2005; Ribeiro et al., 2012), which could reflect differences in the systems utilized or compensatory mechanisms.

#### Antioxidant Proteins and Pathways

In addition to small molecule antioxidants, cells are equipped with an array of proteins and enzymes which scavenge or neutralize reactive oxygen and nitrogen species. These include superoxide dismutases (SOD1), glutathione peroxidases (GPx), peroxiredoxins (PRDXs), and catalase (Sbodio et al., 2018b). The activities of GPx and SOD1, which act on lipid hydroperoxides and superoxide, were decreased in erythtocytes of HD patients (Chen et al., 2007). Cytosolic SOD1 activity was also decreased in the HD parietal cortex and putamen (Browne et al., 1997). Increasing the activity of GPx1, either by genetic or pharmacologic means in cell culture, yeast and Drosophila models of HD mitigated toxicity (Mason et al., 2013). In addition to effects on antioxidant enzymes, mHtt affects the activity of several transcription factors which regulate antioxidant defense and redox signaling pathways. For instance, as explained earlier, mHtt alters SP1 and ATF4 function, thereby affecting expression of CSE and cysteine metabolism (Paul and Snyder, 2014; Paul et al., 2014; Sbodio et al., 2016). Elevated oxidative stress perturbs the compensatory cytoprotective responses in HD. Our studies using a cell culture model of HD revealed that excessive oxidative stress perturbs signaling mediated by ATF4, a master regulator of amino acid homeostasis. Reduction of oxidative stress improves the response of ATF4 to stress stimuli (Sbodio et al., 2016). Nuclear factor erythroid 2-related factor (Nrf2), which orchestrates gene expression pathways which involved in redox balance and proteostasis (Kensler et al., 2007; Tsvetkov et al., 2013; Dinkova-Kostova et al., 2018) is another transcription factor influenced in HD. Blunted Nrf2 signaling was observed in cell culture models of HD (Jin et al., 2013; Rotblat et al., 2014). Thus, stimulation of Nrf2 signaling pathway may be beneficial in HD (Bresciani et al., 2017).

### Mitochondrial Dysfunction

In addition to compromised antioxidant defense as described in the previous section, mitochondrial dysfunction plays a central role in redox imbalance. Mitochondria are the predominant source of ROS and free radicals. Mitochondria are also sites of oxidative phosphorylation (OXPHOS) which is carried out by five multisubunit electron transport complexes (ETCs), designated complexes I–V. Electrons are transported from NADH to NADH coenzyme Q reductase (complex I) to coenzyme Q, which also receives electrons from succinate dehydrogenase (SDH/complex II). Coenzyme Q then relays the electrons to cytochrome C oxidase (complex IV) via complex III (cytochrome bc1). Complex IV reduces molecular oxygen to water using these electrons. Mitochondrial malfunction can lead to oxidative and nitrosative stress through several pathways.

#### Suboptimal Functioning of the Electron Transport Chain (ETC)

**FIGURE 2:**
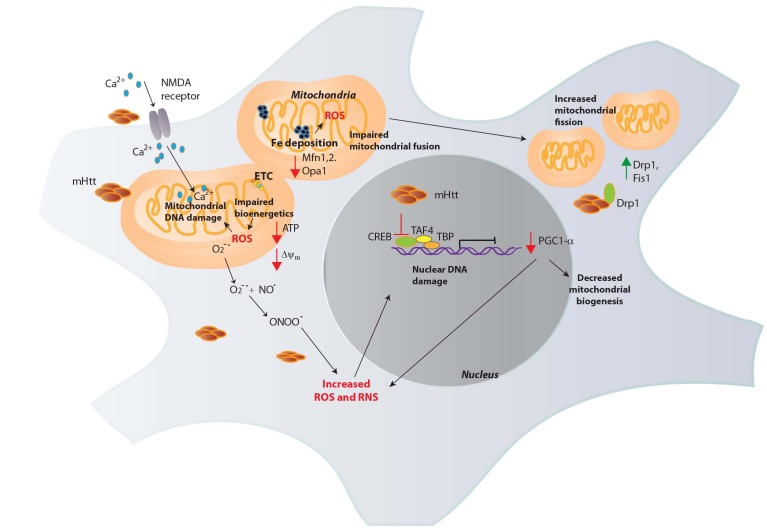
Mitochondrial dysfunction in HD. Mutant huntingtin (mHtt) elicits mitochondrial dysfunction through several mechanisms. mHtt interacts with the outer mitochondrial membrane and could be responsible for several of the observed deficits. mHtt impacts the electron transport chain (ETC), leading to decreased generation of ATP and increased production of ROS such as superoxide (O_2_^-∙^). Increased levels of oxidants damage mitochondrial DNA and nuclear DNA which further compromise mitochondrial function to generate more ROS in a vicious cycle. mHtt also induces defects in Ca^2+^ homeostasis, increasing Ca^2+^ influx via the *N*-methyl-D-aspartate (NMDA) receptors and excitotoxicity. Increased Fe deposition is observed in HD, which can participate in the Fenton reaction to produce the highly reactive OH^∙^, which causes DNA damage. OH^∙^ react with nitric oxide (NO) to generate peroxynitrite (ONOO^-^), which causes nitrosative damage to cellular components. mHtt impairs the transcriptional activity of several transcription factors involved in mitochondrial maintenance and function. The expression of the peroxisome proliferator-activated receptor gamma coactivator 1-α (PGC1-α) transcriptional coactivator, a regulator of mitochondrial biogenesis is diminished in HD by mHtt. mHtt affects the activity of the transcription factor cAMP response element binding protein (CREB) and other proteins such as TATA-box binding protein (TBP) and TBP-associated factor 4 (TAF4), comprising the transcription initiation complex to decrease PGC1-α expression. As PGC1-α has roles in redox homeostasis, its depletion results in oxidative distress. In addition to these effects, mHtt causes mitochondrial fragmentation, binding to the GTPase dynamin related protein 1 (Drp1, whose expression, along with Fis1 is increased in HD) and stimulating its activity to increase fission. Mitochondrial fusion is also impaired in HD due to decreased levels of the proteins involved in fusion, optic atrophy 1 (Opa1) and Mitofusins (Mfn1 and 2).

Mitochondrial malfunction in HD was first implied by nuclear magnetic resonance spectroscopy, which revealed increased lactate levels in the striatum and cortex of HD patients (Jenkins et al., 1993). Activities of complex II, III and IV are decreased in HD brains (Gu et al., 1996; Browne et al., 1997). During the process of respiration, the highly reactive ${\text{O}}_{2}^{•-}$ is generated, especially by complexes I and III (Raha and Robinson, 2000; Murphy, 2009). Electron transport through the complexes is coupled to proton translocation via the mitochondrial inter membrane space, forming an electrochemical proton gradient (electromotive force) and a mitochondrial transmembrane potential (Δψ_m_), necessary for generation of ATP. In HD, the transmembrane potential is perturbed, impacting mitochondrial bioenergetics (Carmo et al., 2018). The close proximity of the ETC to the mitochondrial genome makes it highly susceptible to oxidative and nitrosative damage. Oxidative DNA damage, as measured by levels of 8-hydroxy-2-deoxyguanosine (OH8dG), to mitochondrial DNA (mtDNA) occurs predominantly in the parietal region of the human HD brain, as opposed to the cerebellum or the frontal cortex with the medium spiny neurons (MSNs) accumulating the greatest damage (Polidori et al., 1999). Consistent with these findings, inhibition of complex II by 3-NPA induced HD-like symptoms and neurotoxicity (Beal et al., 1993). mHtt can affect multiple aspects of mitochondrial function which contribute to elevated oxidative stress (Figure 2).

#### Abnormal Ca^2+^ Homeostasis

Mitochondrial Ca^2+^ is perturbed in HD, which impacts diverse mitochondrial dynamics (Kolobkova et al., 2017). mHtt interacts with the outer mitochondrial membrane and affects the mitochondrial permeability transition pore (mPTP), decreasing the Ca^2+^ threshold to trigger mPTP opening, and lowering ATP levels within the organelle (Choo et al., 2004). mHtt has also been shown to interact with Ca^2+^-binding proteins such as calmodulin; disruption of this interaction has proved beneficial (Bao et al., 1996; Dudek et al., 2010). Interaction of mHtt with type 1 inositol (1,4,5)-triphosphate receptor (InsP3R1), an intracellular Ca^2+^ release channel, has also been reported (Tang et al., 2003). Although Ca^2+^ has no direct effect on redox reactions, mitochondrial Ca^2+^ overload induces elevations in ROS levels which in turn cause mtDNA damage (Peng and Jou, 2010; Wang et al., 2013).

#### Impaired Function of Transcription Factors and Proteins Involved in Mitochondrial Function

Mutant huntingtin interacts with and affects the function of a number of transcription factors involved in maintenance of mitochondrial function. For instance, mHtt binds to PPARγ coactivator 1α (PGC-1α), a regulator of several metabolic processes including mitochondrial respiration and biogenesis (Cui et al., 2006). PGC-1α also regulates a battery of proteins involved in antioxidant defense and suppresses the formation of ROS in cells (St-Pierre et al., 2006). In addition, mHtt associates with the PGC-1α promoter and interferes with the transcriptional activation functions of promoter-bound transcription factors, CREB and TAF4 leading to diminished PGC-1α expression, mitochondrial abnormalities, and elevated oxidative stress. Besides these processes, mHtt affects mitochondrial trafficking, fission and fusion, resulting in low ATP levels and impaired bioenergetics (Reddy and Shirendeb, 2012; Carmo et al., 2018). The mitochondrial network in cells is dynamic, undergoing fission and fusion to maintain morphology and function. mHtt interferes with the balance of fission and fusion. mHtt binds to dynamin-related protein 1 (Drp1), whose expression is increased in HD, and enhances its GTPase activity to increase mitochondrial fragmentation and distribution (Costa et al., 2010; Song et al., 2011). Furthermore, nitrosylation of Drp1, which increases its activity, has been reported in HD (Nakamura et al., 2010). In addition, expression of the adaptor protein mitochondrial fission 1 (Fis1) is elevated in HD. Mitochondrial fusion mediated by the proteins mitofusin 1 and 2 (Mfn 1 and 2) and the optic atrophy 1 (OPA1) is also affected, as revealed by decreased levels of these proteins in the striatum and cortex of HD patients (Shirendeb et al., 2011). Accordingly, specifically targeting mitochondrial fission and fusion processes may be beneficial (Yin et al., 2016).

### Autophagy

As discussed above, damaged mitochondria can further exacerbate oxidative stress hence their removal by autophagy can prevent further damage (Murphy, 2009). Autophagy is a conserved lysosomal degradation pathway which acts by clearance of damaged molecules and organelles and mobilizes cellular nutrients in response to several forms of stress (Klionsky and Emr, 2000; Kroemer et al., 2010; Bento et al., 2016; Leidal et al., 2018). Autophagy and oxidative stress have been linked in several studies. There are numerous reports on dysregulated autophagy in HD (Harding and Tong, 2018). Autophagy plays a vital role in maintaining healthy mitochondria in cells by mitochondrial-specific recycling termed mitophagy. Mitochondrial quality control in neurons is highly dependent on autophagy and plays a central role in neuronal bioenergetics (Batlevi and La Spada, 2011; Redmann et al., 2017). As mitochondrial metabolism generates free radicals, autophagic clearance of damaged mitochondria would limit the levels of oxidative and nitrosative damage (Giordano et al., 2014).

### Endoplasmic Reticulum Stress (ER Stress)

Another pathway whose dysregulation leads to elevated oxidative stress is the ER stress signaling cascade. The ER is essential for several cellular functions including but not limited to protein synthesis, folding, maturation, quality control, calcium homeostasis, and glucose metabolism (Almanza et al., 2019). Accumulation of misfolded proteins in the ER triggers a stress response termed the ER stress response, which is a characteristic feature of several neurodegenerative diseases involving protein misfolding and aggregation, such as HD (Hetz and Saxena, 2017). The ER also generates ROS during its normal functions. Expression of N-terminal huntingtin proteins with expanded polyglutamine repeats has been reported to induce cell death in neuronal PC6.3 cell lines that involves endoplasmic reticulum (ER) stress and inhibition of ER stress improved viability and decreased protein aggregation caused by mHtt (Reijonen et al., 2008). One of the proteins involved in the ER stress response is the transcription factor, nuclear factor-κB (NF-κB), which mediates anti-oxidant and anti-apoptotic signaling (Pahl and Baeuerle, 1995; Deng et al., 2004; Sen et al., 2012; Han and Kaufman, 2017). Overexpression of mutant huntingtin proteins in the PC6.3 cell lines results in diminished NF-κB expression and concomitant increase in oxidative stress, decrease in antioxidant levels and enhanced cell death (Reijonen et al., 2010). Accordingly drugs that target the NF-κB pathway may be beneficial in HD. For instance, PRE084, an agonist of the Sigma-1 receptor, Sig-1R, an ER resident chaperone protein, increases NF-κB expression and improves cell viability and mitigates the toxicity mediated by mutant huntingtin proteins in neuronal PC6.3 cells (Hyrskyluoto et al., 2013). Dysregulated NF-κB has also been reported in other studies using RNA-seq and network analysis, where activation has been reported (Miller et al., 2016; Al-Ramahi et al., 2018). Thus, it appears that stage-specific analysis with additional cell types would be necessary to obtain a comprehensive picture of the immune signaling axis in HD.

### Defective DNA Repair

Besides, the processes outlined above, defective DNA repair is linked to elevated oxidative stress and contributes to disease progression. Oxidative DNA damage as assessed by formation of 8-OHdG has been observed in both nuclear and mtDNA (Browne et al., 1997). The expanded CAG repeats in mHtt display both germline and somatic instability; the extent of oxidative damage positively correlates with the degree of expansion (Mangiarini et al., 1997; Kovtun et al., 2007). Expansion of the triplet repeats occurs during repair of DNA damage, especially the oxidized guanosine bases, a process relying on the DNA glycosylase OGG1. This base excision repair, BER, is error prone and leads to expansion of the polyglutamine repeats in a process involving single strand breaks and strand slippage. Expansion of the polyglutamine tract further induces oxidative damage and error-prone repair of the lesions, forming a vicious oxidative cycle (Kovtun et al., 2007). Another DNA repair pathway affected in HD involves the ataxia-telangectasia mutated (ATM) protein (Lu X.H. et al., 2014). ATM initiates repair and is activated during oxidative stress and upon DNA damage (Shiloh and Ziv, 2013). Excessive activation of ATM has been reported in HD; inhibiting its activity delays disease progression in mouse models of HD (Lu X.H. et al., 2014). The N terminal end of normal Htt acts as a sensor of ROS and has been shown to detach from the ER, get phosphorylated and translocate to the nucleus when a methionine residue (M8) is oxidized (DiGiovanni et al., 2016). Htt acts as a scaffolding protein for ATM at sites of DNA damage; the CAG repeats may affect the DNA repair process (Maiuri et al., 2017).

## Exploring Therapeutic Options in HD

A central feature of complex neurodegenerative diseases such as HD is elevated redox stress, which is intimately linked to disease progression. The current understanding of redox signaling and the intricate interplay between the various pathways involved in protection against oxidative and nitrosative stress is not complete. Whether elevated levels of ROS/RNS are the cause or consequence of disease is still not clear, however increased redox stress exacerbates neurodegeneration and blunts cytoprotective responses. Aberrant redox signaling has been observed in other complex disease states such as cancer. While cancer is associated with uncontrolled cell proliferation, neurodegeneration is characterized by premature cell death. Tumorigenesis and neurodegeneration appear to be at two ends of a homeostatic regulatory breakdown. Antioxidant defense mechanisms such as cysteine and glutathione synthesis are bolstered in cancers leading to reduced sensitivity to radio and chemotherapy, whereas the converse appears to operate in most neurodegenerative diseases (Benfeitas et al., 2017; Vucetic et al., 2017; Sbodio et al., 2018b). Thus, targeting cancer cells by increasing ROS levels have proved beneficial in several cancers (Trachootham et al., 2009; Wondrak, 2009; Ishimoto et al., 2011). In neurodegeneration associated with compromised redox homeostasis, diminishing ROS levels may afford therapeutic benefit. Although antioxidant supplementation has been beneficial in mouse models of HD (Table 1), human clinical trials using antioxidants have been largely unsuccessful in HD and other neurodegenerative diseases (Kim et al., 2015). Several factors may underlie the inefficacy of these antioxidants. Most antioxidants used cannot completely intercept or neutralize oxidant species, leading to a slow buildup of oxidative damage. The antioxidants used target only certain species of free radicals and oxidants; a single antioxidant cannot target all oxidant species. Antioxidant therapy can damage normal cellular processes such as autophagy (Underwood et al., 2010). Accordingly, antioxidants that do not negatively interact with normal cellular processes or have off-target effects may be desirable. Reversing or preventing oxidative damage as well as targeting cytoprotective pathways as a whole, as opposed to simple scavenging of oxidants, may afford greater protection against neurodegeneration. Development of non-cytotoxic drugs that possess multitarget, combinatorial selectivity may be more effective (Varshavsky, 1998). Another aspect which has been largely unexplored in HD is the complex interaction between soluble and cellular immune system components in neurodegeneration. In HD, analysis of redox signaling pathways as a function of disease progression is crucial, as end-stage analysis of tissues likely lack the cells of interest which may have already died. Moreover, population studies by themselves may not be sufficient, as individual variations and heterogeneity in redox pathways and genes that control them may affect the treatment outcome. Comprehensive analyses coupling genomics, transcriptomics, proteomics, metabolomics, and clinical data may reveal central hubs for therapeutic intervention. Such approaches are being pursued for cancer therapy and may have applications in HD (Bidkhori et al., 2018). The timing of intervention, the site of delivery and recycling of the antioxidant should also be considered to arrive at effective redox-active molecules. Development of therapies specific for the various reactive oxygen, nitrogen species and other free radicals, would facilitate a greater understanding of disease progression. Combination therapy involving antioxidant supplementation in conjunction with strategies that strengthen cellular antioxidant capacity may also be considered.

**Table 1:** Molecules with antioxidant and neuroprotective effects in HD.

Antioxidant	Systems tested	Effects	Reference
α-Lipoic acid	R6/2 and N171-82Q mice 3-NP model (rat)	Improved survival. Normalized mitochondrial lipid composition, improved mitochondrial structure and mitigated cognitive impairment in 3-NP-treated animals.	Andreassen et al., 2001 Mehrotra et al., 2015
α-Tocopherol (vitamin E)	HD patients 3-Nitropropropionic acid (3-NP) model of HD (rat)	Therapeutic effect on neurologic symptoms for early stage patients. Elevation of creatine kinase (CK) elicited by 3-NP in cytosol was prevented by a combination therapy of vitamin E and Coenzyme Q10 (CoQ10).	Peyser et al., 1995 Kasparova et al., 2006
L-Ascorbic acid (vitamin C)	STHdhQ111 cells, R6/2 mice R6/2 mice STHdhQ111 cells	Modulates glucose uptake via GLUT3, which is compromised in HD. Attenuated the neurological motor signs and behavioral aspects of HD. Improves behavior related ascorbate release. Restored dysregulated amino acid homeostasis modulated by ATF4.	Acuna et al., 2013 Covarrubias-Pinto et al., 2015 Rebec et al., 2003 Rebec et al., 2006 Sbodio et al., 2016
*N*-Acetyl cysteine (NAC)	3-NP model (rat) R6/2 mice R6/1 mice R6/1 mice	Prevented mitochondrial dysfunction, neurobehavioral deficits, decreased oxidative stress. In combination with cysteine supplemented diet, NAC improved motor function, prevented striatal atrophy, and increased survival. Improves mitochondrial function and ameliorates behavioral deficits. Mediated an antidepressant effect. Restored glutamate homeostasis through system ${\text{x}}_{\text{c}}^{-}$	Sandhir et al., 2012 Paul et al., 2014 Wright et al., 2015 Wright et al., 2016
Anthocyanin	R6/1 mice	Modest effects on CAG repeat instability in the ears and cortex.	Mollersen et al., 2016
Coenzyme Q10 (CoQ10)	R6/2 mice R6/2 mice, N171-82Q mice R6/2 mice 3-Nitropropropionic acid (3-NP) model of HD (rat), R6/2 mice HD patients	Increased survival, delayed motor deficits, weight loss, cerebral atrophy, and neuronal intranuclear inclusions. Combination therapy with the NMDA antagonist, remacemide decreased ventricular enlargement, and increased survival. Improved motor performance and grip strength, decreased weight loss, brain atrophy, huntingtin inclusions, and oxidative DNA damage in treated R6/2 mice. Combination therapy with creatine inhibited 3-NP-induced impairment of glutathione homeostasis, decreased lipid peroxidation and oxidative DNA damage. Improved motor function and survival in R6/2 mice. CoQ10 alone is not effective in human trials. Idebenone, an analog, did not significantly alter outcome.	Ferrante et al., 2002 Ferrante et al., 2002 Smith et al., 2006 Yang et al., 2009 McGarry et al., 2017 Brandt et al., 1996
Creatine	Neural progenitor cells HD patients	Increases neural progenitor cell survival in HD. Slows down brain atrophy in premanifest HD. However, was not effective in a randomized controlled trial (CREST-E).	Andres et al., 2016 Bender and Klopstock, 2016 Hersch et al., 2017
Curcumin	3-NP model CAG140 KI Drosophila model of HD	Prevented 3-NP-induced motor and cognitive impairment. Reduced oxidative stress and prevented decrease in succinate dehydrogenase activity. Decreases mutant Htt aggregates in the brain. Partial improvement of transcriptional deficits and behavioral improvement. Decreased photoreceptor neuron degeneration, reduced cell death, and ameliorated motor dysfunction.	Kumar et al., 2007 Hickey et al., 2012 Chongtham and Agrawal, 2016
Cystamine	R6/2 mice R6/2 mice, 3-NP model (mice) YAC128 mice 3-NP model (mice, astrocyte, and mixed astrocyte-neuronal cell culture Drosophila model of HD STHdhQ111 cells	Improved survival, reduced tremor, and abnormal movements and ameliorated weight loss. Increased forebrain cysteine and glutathione levels in R6/2 mice. Protected against 3-NP-induced striatal injury in mice. Prevented striatal neuronal loss, striatal volume loss and striatal neuronal atrophy. Induced Nrf2 and glutathione production. Improved longevity when fed both during larval and adult stages but did not prevent photoreceptor degeneration. When fed to adult flies expressing anti-htt intracellular antibody (intrabody), photoreceptor degeneration was suppressed but longevity effects were absent. Increased mutant cells viability when stressed and prevented ROS formation and increased antioxidant defense.	Karpuj et al., 2002 Fox et al., 2004 Van Raamsdonk et al., 2005 Calkins et al., 2010 Bortvedt et al., 2010 Ribeiro et al., 2013
Deferoxamine	R6/2 mice	Improved motor phenotype.	Chen et al., 2013
Dimethylfumarate	R6/2 mice YAC128 mice	Preserved neuronal morphology. Decreases weight loss and clasping phenotype. Induces the Nrf2 pathway, which maintains redox balance.	Ellrichmann et al., 2011
Isoquercetin	Neurons from *C. elegans* model (128Q) of HD Mutant Htt mouse striatal (109Q) cells	Suppressed the loss of touch response in 128Q worms. Prevented cell death caused by serum deprivation in the 109Q striatal neurons.	Farina et al., 2017
Lycopene	3-NP model (rat)	Improved mitochondrial function and decreased oxidative stress in 3-NP treated animals.	Sandhir et al., 2010
Melatonin	3-NP model (rat) Htt ST14A cells R6/2 mice	Prevents toxicity induced by 3-NP. Decreases lipid peroxidation levels and protein carbonylation. Preserved mitochondrial membrane potential and inhibited cell death pathways in cells. Delays disease onset and mortality in R6/2 mice. Decreased activated caspase-9 and caspase-3 levels.	Tunez et al., 2004 Wang et al., 2011
Monensin	STHdhQ111 cells	Increased survival in response to cysteine deprivation and decreased oxidative stress. Increased the production of the neuroprotective gasotransmitter, hydrogen sulfide.	Sbodio et al., 2018a
Nicotinamide	3-NP model (rat)	Improved motor function and decreased oxidative stress markers (malondialdehyde, nitrites) and increased antioxidant enzyme (glutathione) levels. Decreased lactate dehydrogenase and prevented striatal neuronal death.	Sidhu et al., 2018
Pridopidine	YAC128 mice HD patients	Reversed aberrant gene expression, behavioral deficits, and activated cytoprotective pathways. Activated sigma 1 receptor to reduce oxidative stress and increased BDNF levels. Possible slowing of clinical progression.	Garcia-Miralles et al., 2017; Kusko et al., 2018 Reilmann et al., 2019
Quercetin	3-NP model (rat)	Ameliorated mitochondrial dysfunction, oxidative stress, and neurobehavioral deficits in rats.	Sandhir and Mehrotra, 2013; Amanzadeh et al., 2019
Resveratrol	Human HD lymphoblasts YAC 128 mice	Increased mtDNA copies and mitochondrial-related transcription factors (TFAM and nuclear PGC1α). Increased expression of mitochondrial electron transport chain proteins. Improved motor function.	Naia et al., 2017
Rutin	3-NP model (rat)	Prevented motor and cognitive deficits induced by 3-NP. Prevented striatal degeneration. Decreased oxidative stress.	Suganya and Sumathi, 2017
Selenium	3-NP model (rat) N171-82Q mice	*Bis* selenide prevented weight loss and motor dysfunction. Decreased oxidative stress. Improved motor function, decreased loss of brain weight, decreased mutant huntingtin aggregation and oxidized glutathione levels.	Bortolatto et al., 2013 Lu Z. et al., 2014
Sulforaphane	Neuroprogenitor cell line, HC2S2 harboring EGFP tagged htt exon 1 (28Q, 74Q) 3-NP model (mice) Quinolinic acid (QA) model (rat)	Enhanced mHtt degradation and reduced mHtt cytotoxicity. Stimulated the Keap1-Nrf2-ARE Pathway and Inhibited the MAPKs and NF-κB Pathways to mitigate neurotoxicity. Prevented mitochondrial dysfunction elicited by QA.	Liu et al., 2014 Jang and Cho, 2016 Luis-Garcia et al., 2017
Trehalose	N2A cells harboring tNhtt-60Q–EGFP, tNhtt-150Q–EGFP; R6/2 mice COS-7 and SK-N-SH cells expressing (EGFP-HDQ74); R6/2 mice Human HD fibroblasts	Decreased polyglutamine aggregates in cerebrum and liver, improved motor dysfunction and extended lifespan. Induced autophagy and cleared aggregates. Prevented increase in oxidative stress, ubiquitinated proteins, huntingtin and activated caspase-3 induced by inhibition of proteasome.	Tanaka et al., 2004 Sarkar et al., 2007 Fernandez-Estevez et al., 2014
XJB-5-1-131	HdhQ (150/150) mice R6/2 mice	Diminished oxidative damage to mitochondrial DNA, preserved mitochondrial DNA copy number and function, suppressed motor decline and weight loss, improved neuronal survival. Also effective in animals with well-developed pathology. Promoted weight gain, inhibited neuronal death, decreased neuronal oxidative damage, prevented motor dysfunction or improved it, and reduced a graying phenotype in treated HdhQ (150/150) animals. Reduced weight loss, and improved the motor and temperature regulation deficits, especially in male mice male R6/2 mice. No effect on the lifespan. Slowed somatic expansion at 90 days, and reduced inclusion density.	Xun et al., 2012 Polyzos et al., 2016 Polyzos et al., 2018

*The therapeutic outcome of antioxidant molecules or those mitigating oxidative stress, in cell culture, animal models, and human patients are listed.*

## Concluding Remarks

It is becoming increasingly evident that our knowledge of redox signaling and its bearing on the origin of these complex diseases is not complete. Repurposing/repositioning/recycling/reprofiling drugs, which have cytoprotective effects in the brain, may offer viable options to arrive at safe and effective drugs for these complex diseases. Such strategies are currently actively investigated for cancers and neurodegeneration (Duraes et al., 2018; Turanli et al., 2018). Studies encompassing diverse genetic, epigenetic and environmental factors may facilitate development of specific therapies targeting redox diseases.

## Author Contributions

BP conceptualized the theme of the review. BP and SS wrote the manuscript.

## Conflict of Interest Statement

The authors declare that the research was conducted in the absence of any commercial or financial relationships that could be construed as a potential conflict of interest.
